# abiliti^®^ Closed-Loop Gastric Electrical Stimulation System for Treatment of Obesity: Clinical Results with a 27-Month Follow-Up

**DOI:** 10.1007/s11695-015-1620-z

**Published:** 2015-03-15

**Authors:** T. Horbach, A. Thalheimer, F. Seyfried, F. Eschenbacher, P. Schuhmann, G. Meyer

**Affiliations:** 1 Klinik für Allgemein- und Viszeralchirurgie, Schön Klinik Nürnberg Fürth, Europa-Allee 1, 90763 Fürth, Germany; 2 RoMed Klinik Bad Aibling, Adipositaszentrum, Harthauser Str. 16, 83043 Bad Aibling, Germany; 3 Klinik und Poliklinik für Allgemein- und Viszeralchirurgie, Gefäss- und Kinderchirurgie, Universitätsklinikum Würzburg, Oberdürrbacher Str. 6, 97080 Würzburg, Germany; 4 Zentrum für Adipositas- und Metabolische Chirurgie, Wolfart-Klinik, Ruffiniallee 17, 82166 Gräfelfing, Germany

**Keywords:** Gastric electrical stimulation, Weight loss, Obesity, Implantable stimulation electrodes

## Abstract

**Background:**

The aim of the study was to evaluate the safety and effectiveness of a novel closed-loop gastric electric stimulation device (abiliti^®^ system) featuring a transgastric sensor to detect food intake and an accelerometer to record physical activity to induce and maintain lifestyle changes to treat obesity.

**Methods:**

In a prospective, multi-center study, 34 obese subjects (BMI of 42.1 ± 5.3 kg/m^2^) who passed an eligibility evaluation were implanted with the abiliti system. Safety evaluation included an endoscopic exam to assess the intragastric electrode healing. Efficacy evaluation at 1 year of therapy included weight loss, improvements in eating, and exercise behavior and quality of life.

**Results:**

The transgastric implant controlled by endoscopy was stable for all participants. At 12 months (12 M) the mean excess weight loss (EWL) was 28.7 % (95%CI, 34.5 to 22.5 %), and mean reduction in BMI was 4.8 ± 3.2 kg/m^2^. At 27 months (27 M), the EWL was 27.5 % (95 % CI, 21.3 % to 33.7 %). Eating behavior, evaluated by the “Three Factor Eating Questionnaire”, showed a significant increase in the cognition factor and decrease in the disinhibition and hunger factors at 12 M in comparison to baseline (*p* < 0.001). Participants significantly increased their weekly physical activity (*p* < 0.001). Quality of life was improved in 55.2 % of the patients.

**Conclusions:**

Gastric electrical stimulation with abiliti system in obese participants is well tolerated and leads to significant 12 M weight loss, which was stable to 27 M. We suggest that weight loss is achieved due to the assessed alteration of eating behavior in particular the reduction in disinhibition and hunger, and the measured increase in physical activity.

## Introduction

Morbid obesity is a disease that cannot be successfully treated with conventional lifestyle interventions such as diet therapy or increased physical activity in the vast majority of patients [1–4]. Obesity-related comorbidities necessitate sufficient therapy, with bariatric surgery being considered the “gold standard”, despite the clinically relevant procedure-related morbidity [5, 6]. There is a need for treatment options that reduce surgery related complications and do not produce a permanent change in the gastrointestinal track. One of the options currently under investigation is gastric electrical stimulation (GES). GES has been studied in animal models and also in clinical trials for more than a decade, with conflicting results [7–11]. The abiliti system has been designed with a number of novel features aiming to improve weight loss results. The stimulation parameters are programmable over a wide range in order to induce symptoms and early satiety in all patients. A stimulation testing methodology that uses a visual scale was developed, allowing patients to report their symptom level and adjust therapy to the appropriate level at each follow-up as necessary. In addition, therapy is not delivered continually throughout the day, but is meal-activated, triggered by an intragastric sensor. Finally, the abiliti system combines GES with tools to improve participant eating and exercise behavior, in the form of intake and activity (3D-accelerometer) sensors which provide objective behavior data 24 h a day.

This paper presents the safety and therapeutic efficacy of the first generation abiliti system over a 12-month period (protocol based endpoint) with an additional observational follow-up period offered to all participants and reported here up to the point of 50 % attrition (27 months).

## Research Design and Methods

### Study Design

This was a 12-month prospective, multi-center study conducted in Germany. The protocol was approved by a central ethics committee (FECI 010/1049) and reviewed by each center’s ethics committee. The study was conducted in accordance with Good Clinical Practice and consistent with the Declaration of Helsinki, Informed consent was obtained from all enrolled participants.

### Study Population

The main participant inclusion and exclusion criteria are listed in Table 1. Prior to enrollment, the participants were prescreened using the Three-Factor Eating Questionnaire (TFEQ), which explores the three dimensions of eating behavior: cognitive restraint of eating, disinhibition, and hunger [12]. The test was scored using a proprietary algorithm that characterizes the profile of the highest responders to GES therapy. The participant’s ability to respond to the gastric electrical therapy was assessed with an endoscopic electrical stimulation-based sensitivity screening. The participant was asked to describe their symptoms and to score them on a visual analogue scale (VAS).

**Table 1:** Main inclusion and exclusion criteria

Inclusion criteria	Exclusion criteria
• Age 18 to 60 years • BMI 35 to 55 kg/m^2^ • HbA1c ≤7 % • History of obesity ≥5 years • Women with child-bearing potential (i.e., not post-menopausal or surgically sterilized) must agree to use adequate birth control methods • No significant weight loss (<5 %) within 4 months prior to enrollment • Successful psychological evaluation following the institution psychological evaluation protocol for bariatric surgery • If taking anti-depressant medications, they must be stable for at least 6 months prior to enrollment • Willingness to refrain from using prescription, over the counter or herbal weight loss products for the duration of the trial	• Any prior bariatric surgery • Insulin dependent diabetes • Diagnosed with an eating disorder such as bulimia or binge eating • GI disease such as hiatal hernia (>5 cm), gastroparesis, esophageal motility disorders • Any history of peptic ulcer disease within 5 years prior to enrollment • Arthritis or other pathologies limiting physical activities that physician feels should exclude the participant from the study • Other implanted electrical stimulation devices (e.g. pacemaker, defibrillator, neurostimulator) • Obesity of known endocrine origin

### Therapy System

abiliti® is an implantable system that delivers stimulation therapy triggered by a transgastric intake sensor. The stimulation targets the area of the anterior vagal branches at the lesser curve of the stomach (“crow’s foot”).

Using a programmer and a wand which provides telemetry-based communication with the device, the physician is trained to titrate the stimulation parameters based on the participant’s response and design a participant’s personalized therapy. The parameters of the pulse-train stimulation (4–30 mA, 100–2000 μs pulse duration, 40–120 Hz) are adjusted to obtain the desired symptoms of fullness and satiety, by asking the participant to report their symptom level using a visual scale. The daily therapy is individualized by creating “allowed” periods, where therapy is designed to produce satiety, and “disallowed” periods, where therapy is designed to cause discomfort and stop consumption. The allowed periods are tailored to the participant’s life schedule, to encourage a consistent meal schedule that is preferable for weight loss. Stimulation adjustment is done at initial programming when the stimulation is turned on, and at each subsequent follow-up visit as necessary.

The stimulator stores the meal and activity data derived from the intake sensor and three-axis accelerometer as a daily log, which is uploaded to the programmer and viewed at each follow-up visit. A custom model translates the output of the accelerometer to a daily exercise level and duration.

### Device Implantation

**Fig. 1:**
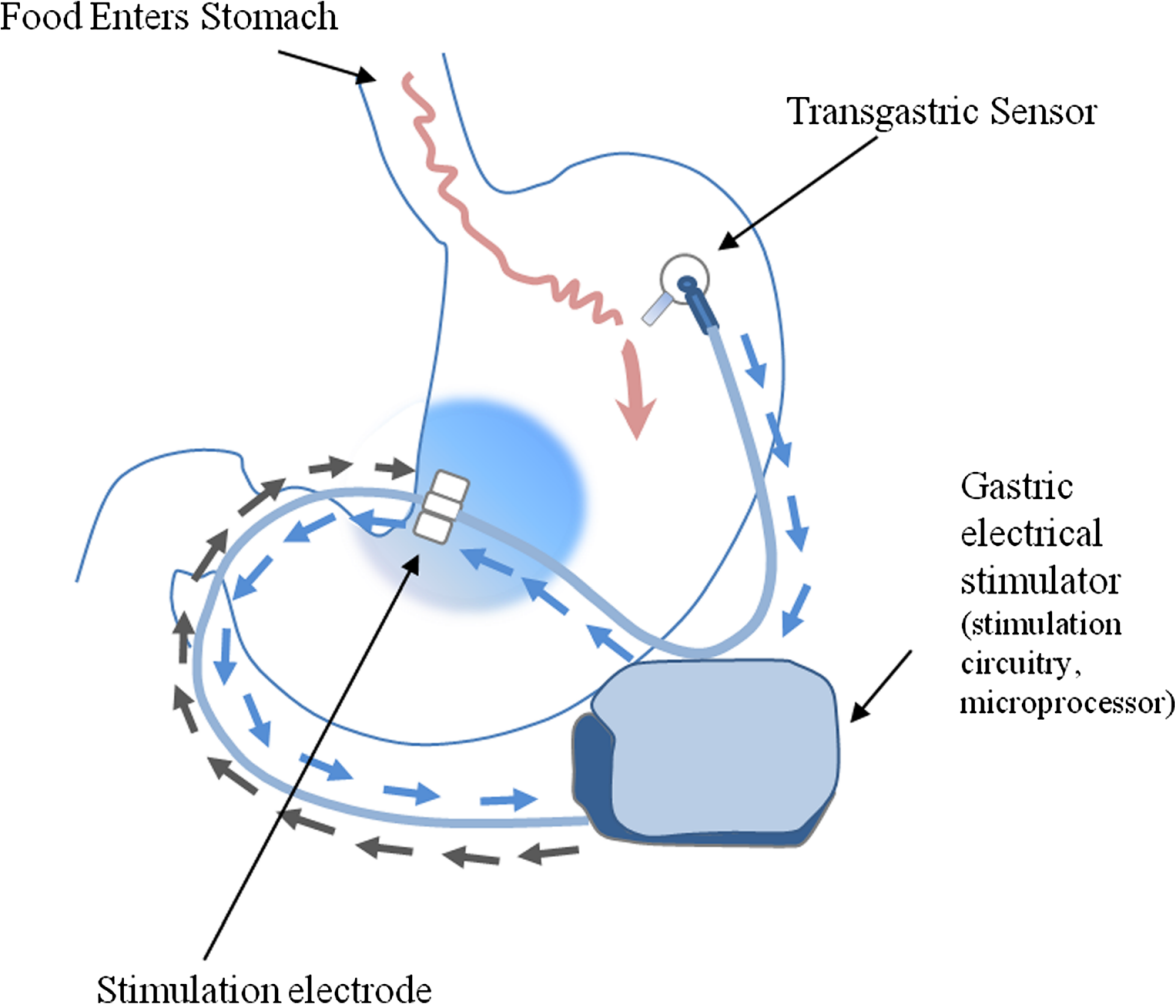
The transgastric sensor detects food entry into the stomach and then triggers the gastric stimulator to deliver therapy at the lesser curvature in the location of the “crow’s foot”

The implantation of the device is performed under general anesthesia similar to other laparoscopic gastric interventions. Three trocars are used for the standardized procedure. The transgastric food sensor is implanted in the anterior wall, body-fundus region, about 3 cm from the greater curvature. The stimulation electrode is implanted in the anterior wall, approximately 4 cm from the gastroesophageal junction and 1.5 cm from the lesser curvature of the stomach, at the point where the Laterjet nerve is divided into three branches (“crow’s foot”). The distance between electrodes should be 3 to 4 cm. Upon inflation of the stomach, a dilating needle is inserted through the trocar which is nearest and perpendicular to the implantation site, to pierce the gastric wall and then place the food sensor electrode with a silicon wafer which is fixed by a seromuscular suture. An upper endoscopy is performed to confirm the intragastric probe extension. A subcutaneous pocket is created in the left upper abdominal quadrant, the lead is exteriorized and connected to the stimulator, which is placed in the pocket. Figure 1 illustrates the placement of the transgastric food sensor, stimulation electrode, and stimulator. The mean duration of the implant surgery was 52.3 min (range 35–110).

### Follow-Up Visits

Two weeks following the implant, the therapy was started, and the meal schedule and therapy parameters were consequently adjusted for each patient. Monthly follow-up was conducted that included weight measurement, dietary counseling and review of food sensor and activity data provided by the device, and if necessary therapy adjustment based on a stimulation sensitivity test.

### Outcome Measures

The primary endpoint was safety of the transgastric implant evaluated by endoscopy examination 3 months after implant. The secondary outcomes were: the frequency and seriousness of all adverse events; the percentage excess weight loss (%EWL) measured at each follow-up visit up to 27 month, assessment of eating behavior with the TFEQ and quality of life with the Moorehead-Ardelt II at baseline and 12 months, and measurement of trends in exercise with the implanted 3D accelerometer during the first 12 months.

### Statistical Analysis

Data were pooled across the study sites and are presented as mean ± SD unless otherwise indicated, comparisons were evaluated using a paired student’s *t* test, and physical activity trends with linear regression. Means, total counts, standard deviations, medians, ranges, and 95 % confidence intervals for the means were calculated for continuous measures. Analyses were performed using Excel version 12 (Microsoft Corp., Mountain View, California, USA) and SAS Version 9.2 software (SAS, Cary, NC, USA).

## Results

### Study Population

One hundred thirty participants were prescreened, 48 % passed the TFEQ criteria and 42 participants consented and were enrolled (Table 2). The disposition of the 42 participants is detailed in Table 3. Table 4 summarizes the population characteristics of the 34 implanted patients.

**Table 2:** Summary of enrollment process at three centers

Site	Investigator	Prescreened *N*	Failed TFEQ *N* (%)	Opted Out *N* (%)	Enrolled *N* (%)
Schwabach	Horbach	28	14 (50)	3 (11)	11 (39)
Wűrzburg	Thalheimer, Seyfried	33	18 (55)	5 (15)	10 (30)
Gräfefing	Meyer	70	36 (51)	13 (19)	21 (30)
	Totals	131	68 (52)	21 (16)	42 (32)

**Table 3:** Disposition of participants—all enrolled participants

	All enrolled abiliti participants (*N* = 42) *n* (%)
Withdrew before surgery	8 (19.0)
Reasons for withdrawal	
Negative eFITT	6 (14.3)
Voluntary withdrawal	1 (2.4)
Investigator withdrawal	1 (2.4)
*Subjects implanted*	*34*
Lost to follow-up <5 months	1 (2.9)
Adverse event (<5 months)	2 (5.9)
*Outcome population*	*31*
Subjects completed the study (12 Months)	31 (100 %)

Italic emphasis signifies that out of the total Subjects Implanted (34), two subgroups, patients who were lostto followup and patients with adverse events were subtracted to obtain the final Outcome population (31)

**Table 4:** Demographics and baseline characteristics of study population

*N* = 34	Mean (SD)	Median	Range (min, max)
Age (years)	43.8 (13.3)	44.0	20, 60
Weight (kg)	117.8 (15.6)	118.0	89.8, 153.7
Excess body weight (kg)	47.4 (14.2)	43.7	25.8,80.6
BMI (kg/m^2^)	41.9 (5.3)	40.6	34.8, 54.3
		*N*	Percentage
Gender	Male	6	17.6
	Female	28	82.4

### Safety Outcomes

A total of 33 gastro-endoscopic examinations of the intragastric probe were performed 3 months after device implant (one participant was explanted for recurrent subcutaneous pocket seroma 1 month after implant). All examinations showed the stability of the probe with a visual estimated measurement of the intragastric extension >10 mm. Good sealing with normal gastric mucosa around the probe was observed and neither overgrowth nor any signs of local infection or erosion were seen. All adverse events (AE) were gathered during the study period. Two AEs were related to the device: abdominal pain due to device position (resolved with repositioning of the stimulator under local anesthesia), and a broken stimulation lead which prevented therapy delivery, and resulted in removal from the study. Two AEs were related to the procedure: one participant presented a severe recurrent subcutaneous pocket seroma without infection, the participant requested the device explant due to discomfort; the second participant developed a post-surgical superficial wound infection which was resolved with antibiotic therapy. There were no intraoperative or serious postoperative complications.

### Weight Loss

**Fig. 2:**
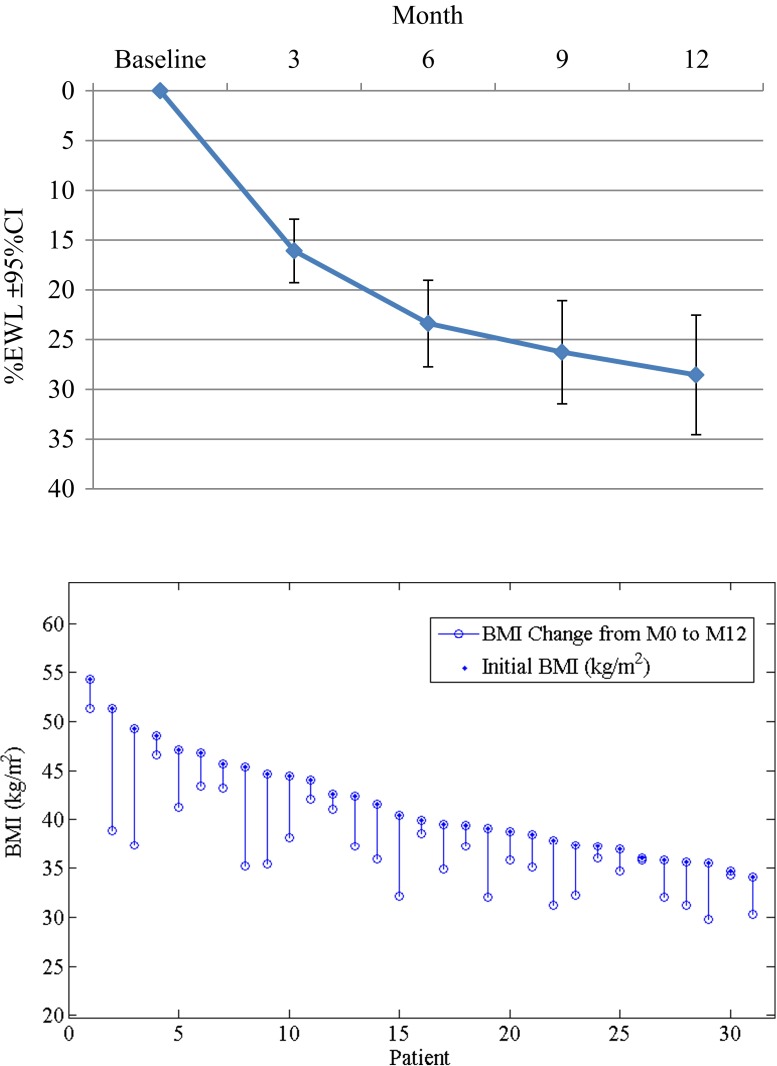
Weight loss outcomes: %EWL at 3, 6, 9, and 12 months (mean ± 95 % CI, *n* = 31), and Individual BMI change at 12 months (*n* = 31) each line segment represents the change in BMI for each subject from baseline to 12 months, showing no correlation between baseline BMI and reduction achieved

**Fig. 3:**
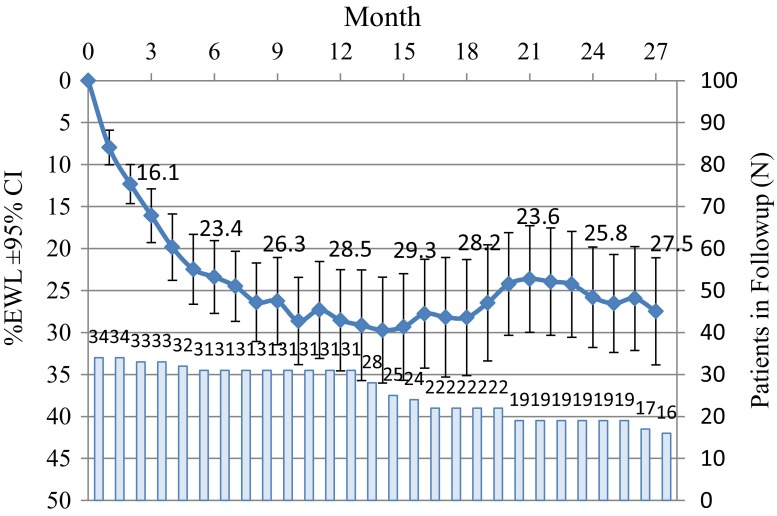
%EWL ± 95%CI throughout the 27 months, with the number of patients remaining in the study at each follow-up indicated with *the corresponding bar*

The participants’ body weight decreased during the entire study period as shown on Fig. 2. The mean %EWL at 12 months was 28.7 % (95%CI, 34.5 to 22.5 %). The average WL was 13.1 kg (95%CI, 16.1 to 9.9 kg) and in BMI was 4.8 ± 3.2 kg/m^2^. No significant correlation was found between baseline BMI and 12 months % EWL (*r* = 0.37). Sixteen participants of the 31 who completed the study remained in follow-up at 27 months. Their weight loss was stable as shown in Fig. 3. The status of the study population is described in Table 5. Due to the early generation technology, premature battery depletion occurred, accounting for some of the attrition seen prior to 27 months.

**Table 5:** Status of study population (*N* = 31) at 27 months

Status	Subjects *N* (%)
Total remaining in follow-up	**16 (52)**
Still implanted, no longer in follow-up (battery depletion occurred and explants were being scheduled (*N* = 5), and participants could not be contacted (*N* = 2))	7 (23)
Explanted (reasons: not satisfied with weight loss (5), interfered with sports (1), battery depletion (1))	7 (23)
Deceased (not procedure- or therapy related)	1 (3)

### Changes in Eating and Exercise Behavior

At 3 months, the TFEQ individual factors analysis showed a significant increase in the cognition factor and decrease in the disinhibition and hunger factors in comparison to baseline measurements (paired *t* test *p* < 0.001). These improvements persisted at 6 and 12 months (*p* < 0.001).

**Fig. 4:**
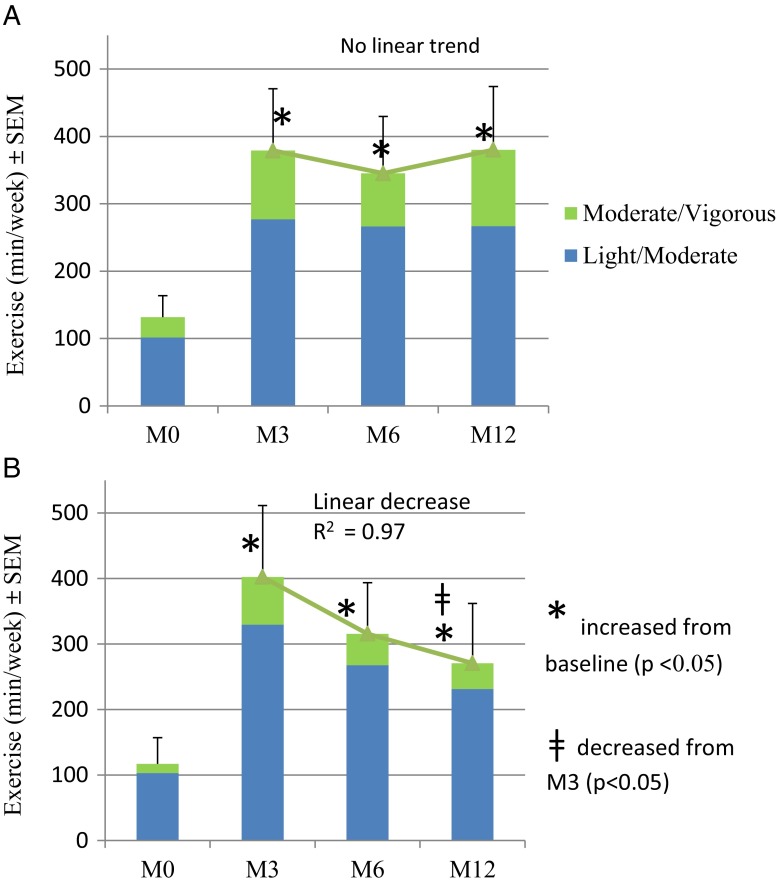
The weekly exercise (min/week) is shown for high performers (**a** EWL ≥ 25, *N* = 18) and lower performers (**b** EWL < 25, N = 13) groups at baseline, 3, 6, and 12 months. The higher performing group exercised consistently between months 3 and 12, at a higher level than baseline, while the low performing group also maintained a higher exercise level compared to baseline, but between M3 and M12 showed a linear decrease (*R* ^2^ = 0.97) in exercise

Compared to baseline, the weekly physical activity duration at 3, 6, and 12 months for all participants was significantly increased (*p* < 0.001), as shown in Fig. 4a, b. However, the patients who lost less weight (%EWL < 25) showed a linear decrease in weekly exercise after M3 (*R* ^2^ = 0.97), with an average decrease of 132 min/week between M3 and M12 (*p* < 0.05).

### Quality of Life

**Fig. 5:**
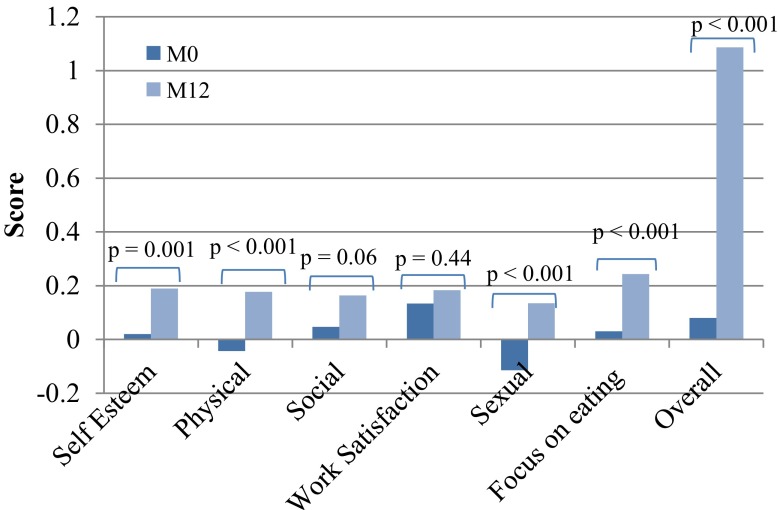
Change in overall QOL score and key area scores from baseline to 12 months. From baseline to 12 months, the overall quality of life improved for 16 subjects (55.17 %), remained the same for 10 (34.48 %) and declined for 3 (10.34 %). Analyzing individual key area scores, four areas were significantly improved at 12 months, self esteem, physical, sexual, and focus on eating (*p* < 0.001)

The quantitative analysis showed a mean increase of 1.0 point for all participants at 12 months compared with their baseline values. This increase represented a qualitative change in category from “Fair” to “Good”. The individual key area scores were also analyzed, and four areas significantly improved at 12 months (Fig. 5).

## Discussion

This prospective study confirms the safety and efficacy of the abiliti system in the treatment of obesity over a period of 27 months, but a longer-term study is needed. The use of a transgastric probe was shown to be safe as confirmed by endoscopic examination. The mean duration of surgery and invasiveness was comparable to the placement of a gastric band [13]. Compared to other bariatric surgeries reporting gastric symptoms including reflux and vomiting [14], GES treatment was well tolerated by the participants with no adverse reactions noted due to the programmed stimulation therapy. The WL results published by the nonrandomized GES studies previously performed in Europe and USA ranged from 21 to 23 % EWL [9, 15]. This study shows an improvement in outcome (28.7 % (95%CI, 22.5 to 34.5 % EWL)).

The overall quality of life for all participants significantly improved when comparing baseline to 12 months, these results are consistent with other successful surgical WL therapies [16, 17]. The Moorehead-Ardelt QOL II questionnaire has been shown to be well correlated with widely used health and well-being indicators [18], the score has a positive correlation with WL, but is not a good indicator of digestive side effects [19].

The changes in the participants’ eating behavior as assessed by the TFEQ show that abiliti therapy improves self-awareness and confidence in their ability to control their eating and decreases hunger for all participants. These findings are consistent with the results from weight loss studies using surgical and behavioral therapy [20–23]. The significant reduction in hunger supports the enhancement of satiety as a WL mechanism with GES therapy.

The effect of the system’s ability to modify eating behavior is seen in the stable WL of participants that remained in long-term follow-up. The relatively large percentage of patients remaining in follow-up for more than 2 years, compared to a 30 % attrition rate at 1 year for pharmacotherapy [24] is evidence of patient satisfaction with the combination of stable weight loss and high quality of life achieved with the positive behavioral changes promoted by the system This is in contrast to the drastic lifestyle modifications required with other bariatric procedures [25]. Variability in weight loss results can be observed in clinical studies regardless of the treatment applied, behavioral, or surgical [6, 26], suggesting many factors affect weight loss outcomes; including psychological, behavioral, and environmental factors. To minimize these factors, an arduous screening process was applied in the control randomized SHAPE trial [10] (4802 candidates screened, 190 participants enrolled). This process did not improve the study outcome possibly because it identified a control group highly selected to succeed with a low-calorie diet and a monthly support group. In the abiliti study, the TFEQ was chosen as a prescreening process to limit the population to participants with a high “cognitive restraint” factor—which has been shown to be particularly important in the successful treatment of obesity—and a high hunger factor—because they may benefit from a treatment which enhances satiety [12]. In addition, a participant’s response to GES therapy will differ based on their specific neural anatomy and stimulation response threshold. Yao [27] showed that the inhibitory effects of gastric stimulation were correlated with the visceral sensitivity of the participant to gastric stimulation. The endoscopic stimulation test was designed to test patient sensitivity to electrical neural stimulation of the gastric wall before performing surgery, and 14 % of the participants were screened out because they lacked the required response. The type of symptoms observed were a feeling of satiety or fullness for 42 % of the participants, gastric pressure for 58 %, and nausea for 16 %.

The mode of stimulation may partially explain the improvement in WL compared to other GES systems, since the other stimulators provided continuous stimulation below the participant’s symptomatic threshold. Continuous stimulation may engender a neuromuscular adaptation phenomenon [28] that could be responsible for loss of efficacy. Furthermore, asymptomatic stimulation may not be enough; the early sensation of satiety and fullness may be ignored by some participants. GES that is adapted to the individual and programmed to be above sensation threshold has been shown to be effective in a recent animal study [29]. Gastric banding and other obesity surgical procedures penalize the participants when they are overeating (e.g., pain, nausea, vomiting), and these disagreeable symptoms force the participant to maintain appropriate eating behavior. But these penalties can also lead to the creation of aberrant eating habits.

The abiliti system has both an activity and a food intake sensor which can provide objective sensor-based feedback which has been shown to promote behavioral modification [30–32]. Using the activity data provided by the device, the clinician was able to advise and encourage the patient towards reaching their exercise goals, resulting in the observed increase in total weekly exercise duration. The study results show a progressive increase in exercise duration and intensity throughout the follow-up period for the participants with higher WL, confirming the importance of exercise in long-term weight loss [33]. In addition to triggering the therapy, the food intake sensor also provides important behavioral feedback regarding eating time and frequency, which helps the clinician identify patterns [34] and focus on solving issues with regards to an individual patient’s eating habits.

This study is limited due to the lack of a control group. A blinded control group is challenging because the abiliti therapy combines both GES and sensor-based behavior feedback. We expect that improvement in patient behavior with sensor-based feedback is due to a combination of increased self-awareness, and awareness that their behavior is being monitored.

## Conclusion

In this prospective study, GES proved to be safe with promising treatment results. The abiliti^®^ system offers a weight loss therapy option that has the advantage of no permanent anatomic changes. The study has shown that the abiliti system creates change in both eating and exercise behavior which leads to sustained weight loss. Guided by behavior change theories such as presented by Prochaska et al. [35], clinicians of different obesity treatment disciplines could use the sensor data provided by the abiliti system to effectively promote long-term adherence to exercise and diet guidelines. These results warrant additional controlled clinical studies to confirm long-term safety and efficacy of the therapy system.
